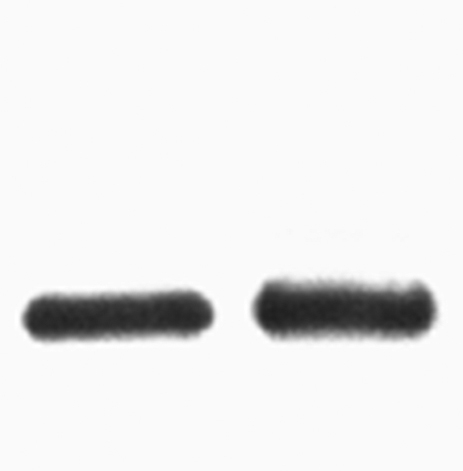# Corneal keratin aggresome (CKAGG) formation and clearance by proteasome activation

**DOI:** 10.1016/j.heliyon.2018.e01012

**Published:** 2018-12-08

**Authors:** Fawzia Bardag-Gorce, Andrew Makalinao, Imara Meepe, Richard H. Hoft, Daileen Cortez, Joan Oliva, Amanda Laporte, Jeremy Stark, Amber Gorce, Michael Di Lorenzo, Samuel W. French, William Lungo, Yutaka Niihara

**Affiliations:** a Los Angeles BioMedical Research Institute, Torrance, CA, 90502, USA; b Emmaus Medical, Inc. 21250 Hawthorne Blvd. Suite 800, Torrance, CA, 90503, USA

**Keywords:** Cell biology, Ophthalmology

## Abstract

**Purpose:**

To understand the mechanism of corneal keratin expression and clearance in corneal epithelium with Limbal Stem Cell Deficiency (LSCD). The hypothesis is that LSCD-induced proteasome dysfunction is a contributing factor to keratin aggregation, causing corneal keratin aggresome (CKAGG) formation.

**Method:**

LSCD was surgically induced in rabbit corneas. LSCD corneal epithelial cells (D-CEC) were collected to investigate keratin K4 and K13 expression and CKAGG formation. Oral mucosal epithelial cells (OMECS) were isolated and cultured to study K4 and K13 expression. Cultured cells were treated with proteasome inhibitor to induce CKAGG formation.

**Results:**

K4 and K13 were strongly expressed in D-CEC, with additional higher molecular weight bands of K4 and K13, suggesting CKAGG formation. Double staining of K4/K13 and ubiquitin showed co-localization of these keratins with ubiquitin in D-CEC. Proteasome inhibition also showed K4/K13 modification and accumulation in cultured OMECS, similar to D-CEC. Proteasome activation was then performed in cultured OMEC. There was no accumulation of keratins, and levels of unmodified keratins were found significantly reduced.

**Conclusion:**

Results showed an abnormal expression of K4 and K13 after LSCD-induced proteasome dysfunction, which coalesce to form CKAGG in Corneal Epithelial Cells (CEC). We propose that CKAGG formation may be one of the causative factors of morphological alterations in the injured corneal epithelium, and that CKAGG could potentially be cleared by enhancing proteasome activity.

## 1 Introduction

Corneal epithelial cells are characterized by the expression of keratin K3/K12 pair; conjunctival epithelial cells are characterized by the expression of K4/K13 [1]. Corneal epithelium is constantly renewed from basal cells and from limbal stem cells [2, 3]. In case of Limbal Stem Cell Deficiency (LSCD), corneal epithelium keratin expression shifts from K3/K12 to K4/K13 positive cells, indicating conjunctivalization of central cornea [4, 5]. Conjunctival cells have been the subject of several research studies, because not only they are a useful diagnostic tool to evaluate the ocular surface disorders [6, 7], but they also are a source of cells to reconstruct corneal surface in cell-based therapy approaches for the treatment of LSCD [8, 9]. To our knowledge, there have been no reported studies investigating the expression of conjunctival keratins in corneal epithelium after injuries.

It is well known that when the limbal barrier is absent, conjunctival cells invade the central cornea, showing K4/K13 positive staining, conjunctivalization, and eventually corneal opacification [10, 11]. Presence of K13 and goblet cells has generally been considered evidence for the overgrowth of conjunctival epithelium onto cornea in the early phase of the limbal stem cell deficiency models [12, 13]. Studies have shown that, while Muc5ac expressing cells tend to disappear from central cornea, K13 expressing cells persist on corneal surface and some of the conjunctival epithelial cells transdifferentiates into cornea-like cells [14, 15]*.* However, it is not clear whether K13 expression in corneal epithelial cells with LSCD is similar to that in conjunctival epithelial cells. In addition, K4 expression has not been reported as a conjunctival biomarker for the central cornea in late phase of animal models of LSCD.

Proteasome dysfunction is known to be a major cause of intermediate filament collapse and aggresome formation. Constitutive proteasome (CPR) dysfunction has been shown to cause keratin aggregation and accumulation, in both human and animal cells [16, 17], indicating the important role of CPR in regulating epithelial cell keratin clearance. It is our hypothesis that, after cornea injury, both keratins K4 and K13 undergo post-translational modifications and aggregate to form corneal keratin aggresomes (CKAGG) due to the failure of clearance by CPR. In previous study [18], CPR dysfunction was found in corneal epithelial cells in a rabbit model with LSCD; the inflammation caused by surgically-induced LSCD resulted in an increase in immunoproteasome (IPR) formation, at the expense of CPR, causing a slowdown in ubiquitinated protein clearance.

IPR formation is driven by high levels of cytokine production, such as interferon gamma (IFNg) and TNFα [19, 20, 21]. Consequently, altered and unwanted proteins, such as modified keratins, accumulate to aggregates and form aggresomes. In the present study, we used corneal epithelial cell lysates from rabbits with experimentally-induced LSCD [4] to investigate CKAGG formation and clearance. Corneal epithelial cells with LSCD were compared to healthy corneal and conjunctival epithelial cells to measure keratins expression. A double staining of keratins and ubiquitin indicated co-localization of keratins with ubiquitin, suggesting that keratins might have been ubiquitinated to be cleared by the proteasome. To test our hypothesis, rabbit Oral Mucosal Epithelial Cells (OMEC), which are normally characterized by K4/K13 expression—similar to conjunctival epithelial cells (CjEC), were used in an *in vitro* study. OMEC were isolated from rabbit buccal biopsies, cultured and treated with a high dose of proteasome inhibitor to induce CKAGG formation. Results demonstrated that, when proteasome was significantly inhibited, keratin levels were up regulated, leading to keratin aggregates accumulation. However, when proteasomes were activated, there was no keratins accumulation and unmodified keratins levels were down regulated. These results suggest that proteasome activation may have a therapeutic effect for ocular surface injuries, such as LSCD, thermal burns, chemical burns, and may possibly improve corneal transparency.

## 2 Material and methods

### 2.1 Rabbit model of LSCD

New Zealand white rabbits weighing 2.5–3 kg were used. The rabbits were caged in an AALAAC-accredited facility located on the campus of the Los Angeles Biomedical Research Institute at Harbor-UCLA Medical Center. A full-time veterinarian who specializes in laboratory animal medicine overseen their care. Caging and husbandry was provided according to the guidelines of the NIH publication, “Guide for the Care and Use of Laboratory Animals”. The protocols for animal use was approved by the Institutional Animal Care and Use Committee (IACUC) of the Los Angeles Biomedical Research Institute. Total LSCD was surgically induced in rabbits as previously reported [4]. Briefly, rabbits were sedated, subjected to 360° lamellar limbectomy, and followed for 3 months until LSCD was confirmed stable by the ophthalmologist.

### 2.2 Corneal epithelial cells (CEC) sampling

Healthy CEC (H-CEC) were sampled before lamellar limbectomy, and D-CEC were sampled 3 months after the surgical creation of LSCD with lamellar limbectomy. At month 3, rabbits were lightly sedated and corneal epithelial cells were sampled by exposing the corneas to 20% isopropyl alcohol for one minute to loosen up the corneal epithelium. The corneas were then washed 3 times with saline and CECs were harvested from the corneal surface using a crescent knife 2 mm angled, double bevel (Katena products, Inc; Denville, NJ). CEC were suspended in PBS, centrifuged and re-suspended in lysis buffer containing 20 mM Tris-HCl pH = 7.5; glycerol 10% EGTA 1 mM; DTT 1 mM; protease and phosphatases inhibitor cocktail as instructed by the supplier (Sigma, St Louis, MO). Cell membranes were disrupted by 5 second sonication at 4 °C. Samples were then frozen at -80 °C until all samples (H-CEC and D-CEC) were gathered for biochemical analysis.

### 2.3 Western blot analysis

Protein concentration was measured using Bio-Rad protein assay. Two μg of total protein from sampled and lysed corneal epithelial cells and from harvested CAOMEC were separated by SDS-PAGE electrophoresis using 124-20% gradient polyacrylamide gels. Proteins were transferred to a PVDF membrane (Bio-Rad, Hercules, CA) for 1 h in 25 mM Tris-HC1 (pH = 8.3), 192 mM Glycine and 20% methanol. Membranes were probed with primary antibodies against K4, K13 and ubiquitin (Santa Cruz Biotechnology, Santa Cruz, CA), Fk2 for mono and polyubiquitinated proteins (Enzo Life Sciences, Inc. Farmingdale, NY), beta actin (Sigma). Goat anti-mouse and sheep anti-Goat antibodies (Bio-Rad, Hercules, CA) were used as secondary antibodies. Immuno-detection was performed using ECL plus (Amersham Bioscience Corp., Piscataway, NJ). Bands densitometric measurements were performed using the GS-800 imaging densitometer (Bio-Rad, Hercules, CA).

### 2.4 Immunohistochemistry

Corneal tissue from healthy rabbits and rabbits with LSCD were fixed in 10% neutral buffered formalin. Processed tissues were paraffin embedded, sectioned, and slides were used for immunofluorescent stainings. Mouse monoclonal antibodies against keratins K4 and K13 (Santa Cruz Biotechnology CA), goat polyclonal against ubiquitin (Santa Cruz Biotechnology) were used in single and double staining. K4, K13 and ubiquitin were detected with second antibody conjugated to Alexa Fluor® 488. Ubiquitin was detected with second antibody conjugated to Alexa Fluor® 568 donkey anti-goat. 4′,6-diamidino-2-phenylindole (DAPI) (Thermo Scientific, Waltham, MA) was used for nuclear staining. The level of protein expression was imaged using a Nikon 400 fluorescent microscope with FITC cube and a triple band cube to detect FITC, Texas Rex and DAPI. Picture processing and analysis was performed using Adobe Photoshop CS5.

### 2.5 Transmission electron microscopy analysis

Corneal epithelial cells were collected from the surface of rabbit corneas with LSCD. Cells were centrifuged to produce pellets that were fixed in 2.5% glutaraldehyde and in 0.1 M sodium cacodylate buffer at 4 °C overnight. Subsequently, pellets were post-fixed in 1% osmium tetroxide for 1 hour. After dehydration, specimens were embedded in Epon 812 epoxy resin. Ultra-thin sections were stained with 3% uranyl acetate and lead nitrate, and examined with a HITACHI H-600 electron microscope at 75 kV. LSCD-diseased and normal corneal epithelial cells were analyzed.

### 2.6 Oral mucosa epithelial cell isolation, cell culture and treatment with proteasome inhibitor

Oral mucosal biopsy was performed on a sedated animal using a 6 mm diameter disposable punch biopsy instrument (Biopunch HealthLink, Jacksonville, FL). The biopsy specimen was washed in sterile saline, sanitized in povidone iodine, washed again in sterile saline and then washed in DMEM cell culture media. The specimen was then used to isolate oral mucosal epithelial cells, as previously described [22]. Briefly, the specimen was minced and incubated with Dispase I for one hour at 37 °C (Roche Diagnostics GmbH, Mannheim, Germany), the epithelium was separated from the *lamina propria* and subjected to trypsin digestion to separate the epithelial cells. The isolated primary epithelial cells were seeded at 5×10^5^ density on Transwell insert (Corning Inc.), and co-cultured with mitomycin C (MMC)-treated NIH/3T3 feeder cells. After 4 days of cell culture, the media was changed and EGF was added at 10 ng/mL final concentration. Cell culture media was changed every two days. When the cells formed a multilayered cell sheet, proteasome inhibitor (PS341, Bortezomib/Velcade®, Millennium Pharmaceutical) was added in cell culture media for 24 h and for 72 h (at 2.5, 5, 10, 20, and 50 nM), to, respectively, achieve proteasome inhibition and proteasome activation.

### 2.7 Proteasome activity

One μg of total protein from sample homogenates was used. The reaction mixture contained 50 mM Tris–HCl pH = 8, 1 mM DTT, and 40 μM Suc-Leu-Leu-Val-Tyr-AMC (Sigma Aldrich) substrate for chymotrypsin-like activity or 40 μM of Boc-Leu-Ser-Thr-Arg-AMC for trypsin-like activity or 100 μM of Ac-Gly-Pro-Leu-Asp-AMC (Sigma Aldrich) for Caspase-like activity. 5 mM ATP was added to the reaction mixture to distinguish 26S proteasome activity from 20S proteasome activity. The mixture was then incubated for 30 min at 37 °C, and stopped by adding 100 μM monochloroacetate and 30 mM sodium acetate pH = 4.3. Fluorescence was determined by measuring the release of AMC (λ excitation: 370 nm, λ emission: 410 nm), using a Perkin Elmer LS 30 spectrofluorometer.

### 2.8 Statistics

Data were obtained from at least three separate experiments. Bars represent mean values ± SEM. One-way ANOVA and Student–Newman Keuls analysis determined P values for multiple group comparisons (Sigma-Stat softdish, San Francisco, CA). Statistical significance is set at p = or < to 0.05. Bar graphs were shown as Mean ± SEM, n = 3–4.

## 3 Results

**Fig. 1:**
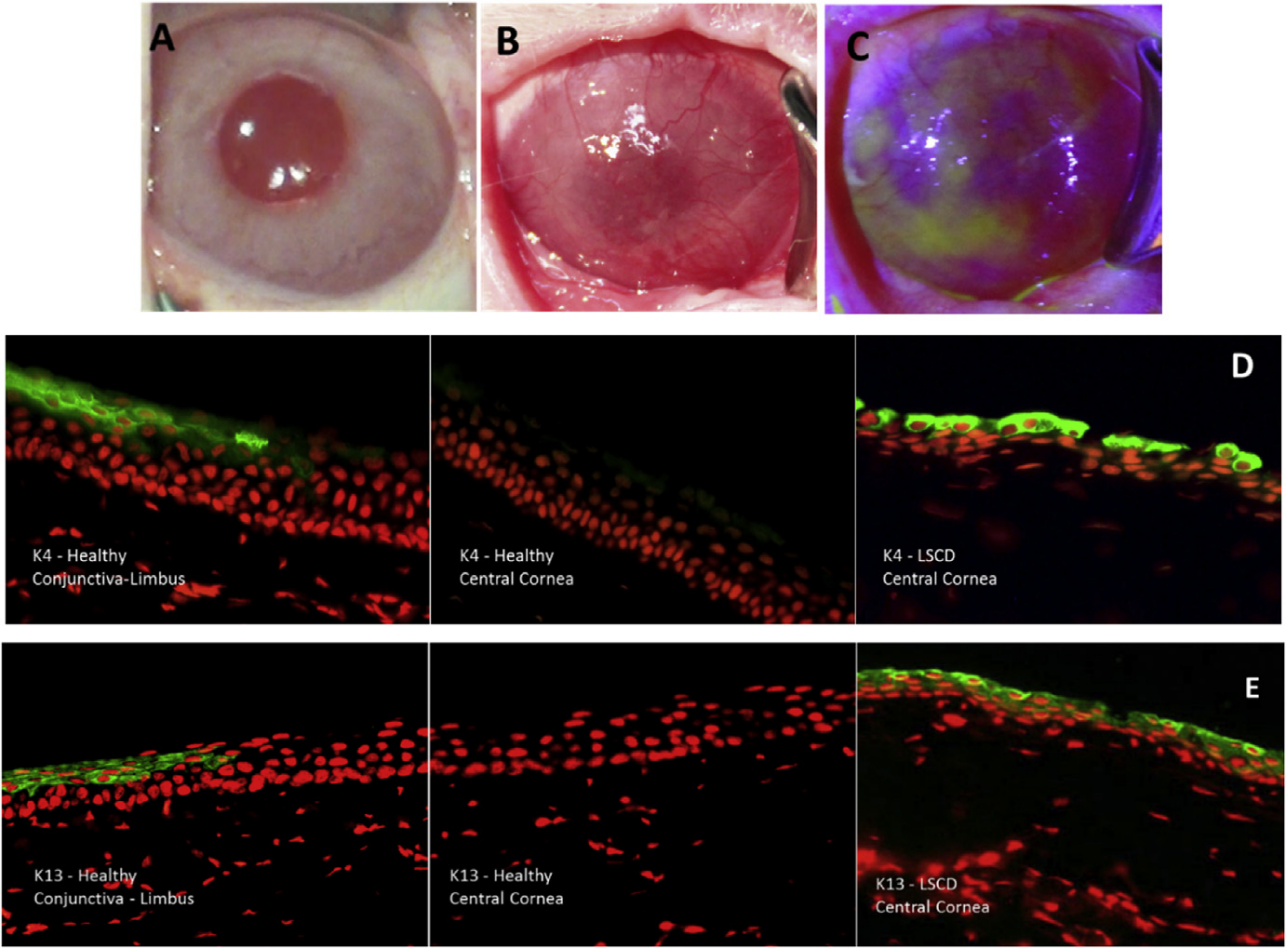
A: is rabbit healthy eye. B and C: is rabbit LSCD-diseased eye. C is the same eye stained with fluorescein. Note that cornea is opaque. D and E: are respectively K4 and K13 expression, analyzed with immunofluorescent staining in green (red is nuclear staining with propidium Iodide, 40x). Note that both K4 and K13 stained positive in healthy conjunctiva-limbus area and negative in central cornea. However, both K4 and K13 stained positive in LSCD central cornea.

LSCD was surgically induced by a 360 lamellar limbectomy, as previously reported [4]. Corneas were medically managed for the first month after limbectomy and followed for two months until the limbectomy-induced pathology was stable. Fig. 1 B and C show LSCD-induced complete conjunctivalization of the cornea. Keratin distribution was investigated in healthy and LSCD-diseased corneas. Fig. 1 D and E show that both K4 and K13 were detected in the healthy conjunctiva-limbus area, but were not detected in central cornea. However, K4 and K13 were detected in conjunctiva-limbus area and in central cornea in LSCD case (Fig. 1 D and E).

Results also show that the limbus stained positive for K4 and K13 in healthy eyes. When the limbus was absent, the conjunctival cells invade central cornea.

**Fig. 2:**
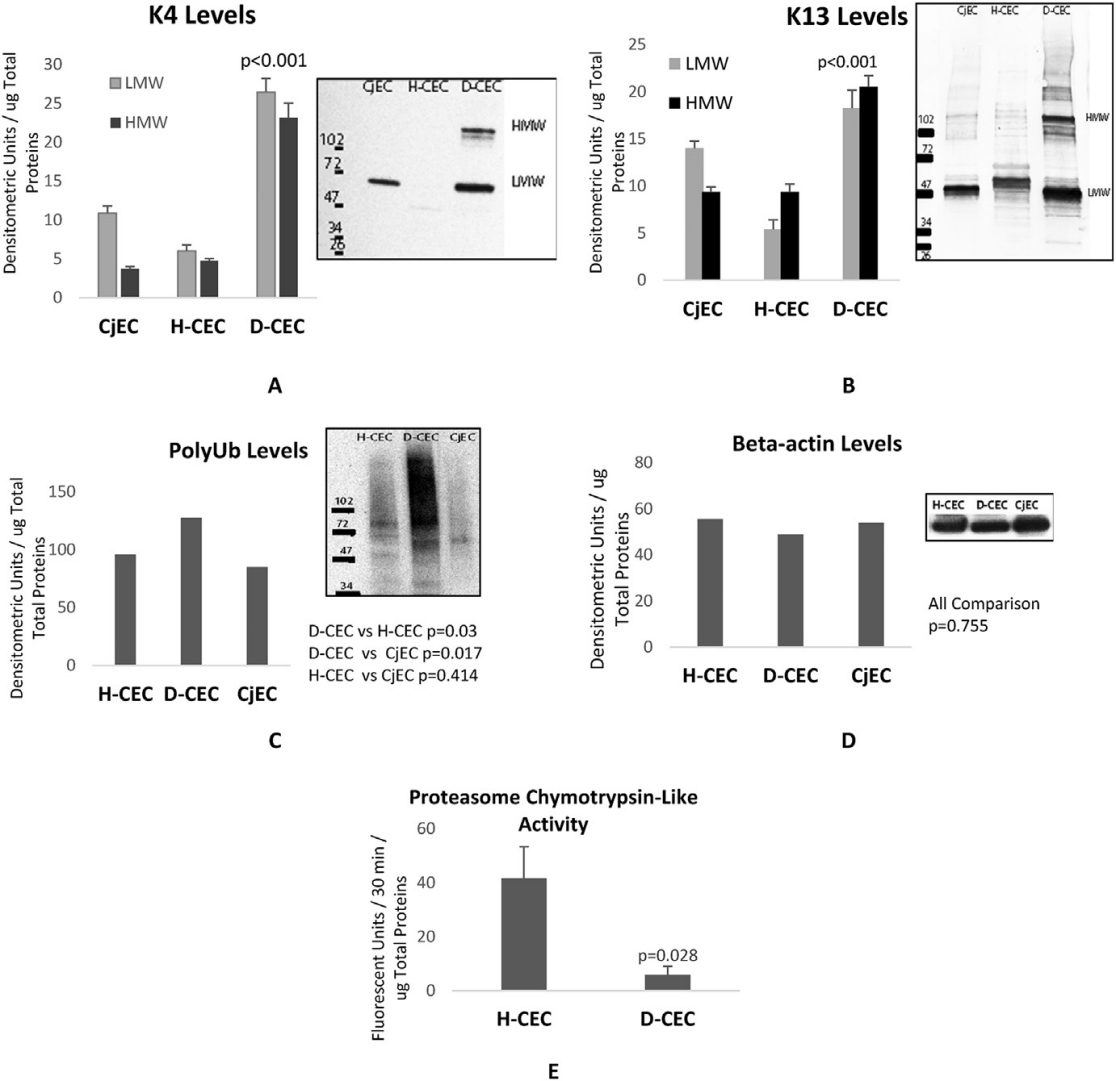
Biochemical analysis of LSCD corneal epithelial cells (D-CEC) sampled from LSCD's corneas showed that K4 and K13 levels were significantly increased compared to healthy CEC and conjunctival CEC (CjEC) (A and B). High molecular weight of K4 and K13 proteins were also detected reflecting an insoluble form of keratin aggregates of K4 and K13. C: shows that poly ubiquitinated proteins levels were significantly high in D-CEC indicating that D-CEC proteins were ubiquitinated and accumulated in insoluble aggregates. D: shows beta actin levels as an indication of loading control for each sample. E: shows proteasome chymotrypsin-like enzyme activity (Mean ± SEM, n = 3).

The semi-quantitative proteomic analysis of CEC sampled from healthy and LSCD-corneas (H-CEC and D-CEC respectively) showed that K4 and K13 expression were significantly increased in D-CEC when compared to H-CEC (Fig. 2 A and B, LMW). We also found an accumulation of K4 and K13 with extra bands at high molecular weight in D-CEC (Fig. 2 A and B, HMW), suggesting a failure in the clearance of modified keratins, and an aggregation of K4 and K13 that is deposited in insoluble form without clearance. We called these corneal keratins aggresome CKAGG.

A higher level of poly-ubiquitinated proteins in D-CEC, compare to H-CEC (Fig. 2 C), was an indication of the accumulation of ubiquitinated proteins tagged for degradation by the proteasome. It also indicated that there was a decrease in proteasome enzyme activity (as shown in Fig. 2E), which confirmed our previously reported decrease in all three constitutive proteasome (CPR) activities [chymotrypsin-like, trypsin-like and caspase-like activity] in LSCD-diseased CEC [18]. This decrease in CPR activity is proposed to be the *cause of the accumulation of unprocessed keratins and the formation of keratins aggresome in LSCD-CEC*.

**Fig. 3:**
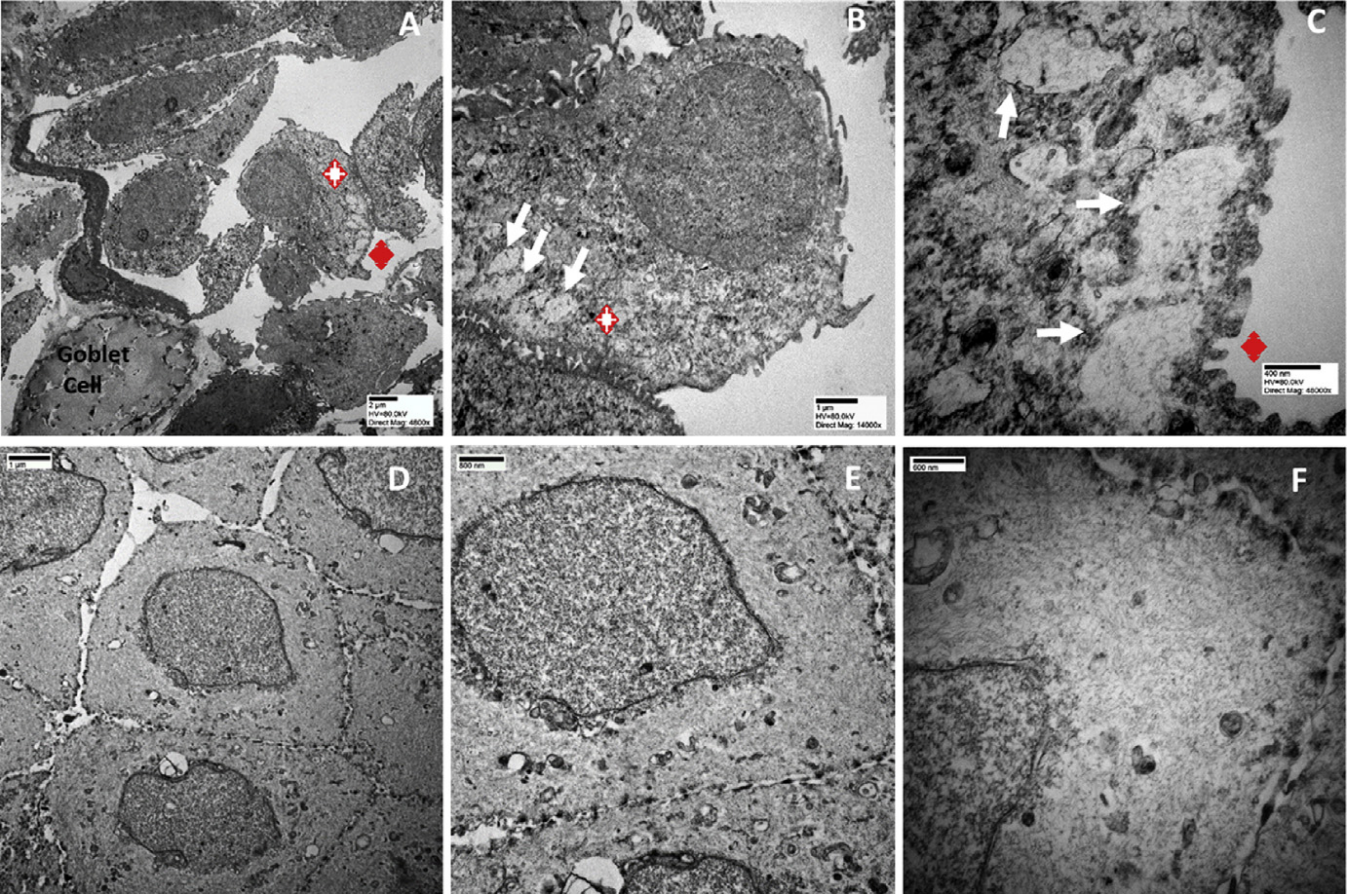
Transmission Electron microscopy analysis of LSCD-diseased (A to C) and healthy corneal epithelial cells (D to F).

Corneal epithelial cells were sampled from LSCD-diseased and healthy ocular surface. The cells were suspended in lysis buffer and were centrifuged for 30 min at 14 k rpm to sediment all the cells. The obtained pellets were prepared for transmission electron microscopy analysis. Fig. 3 shows that LSCD-diseased cells were abnormal with material deposition in the cytoplasm resembling protein aggregation (Fig. 3 A to C). The healthy cells (Fig. 3 D to F) showed the usual cubical shape of an epithelial cell. Higher magnification of the cytoplasm material from the LSCD diseased cell (Fig. 3 B and C) shows a granular pattern, as compared to the normal cell (Fig. 3 E and F), in which the cytoplasm contained filamentous material.

**Fig. 4:**
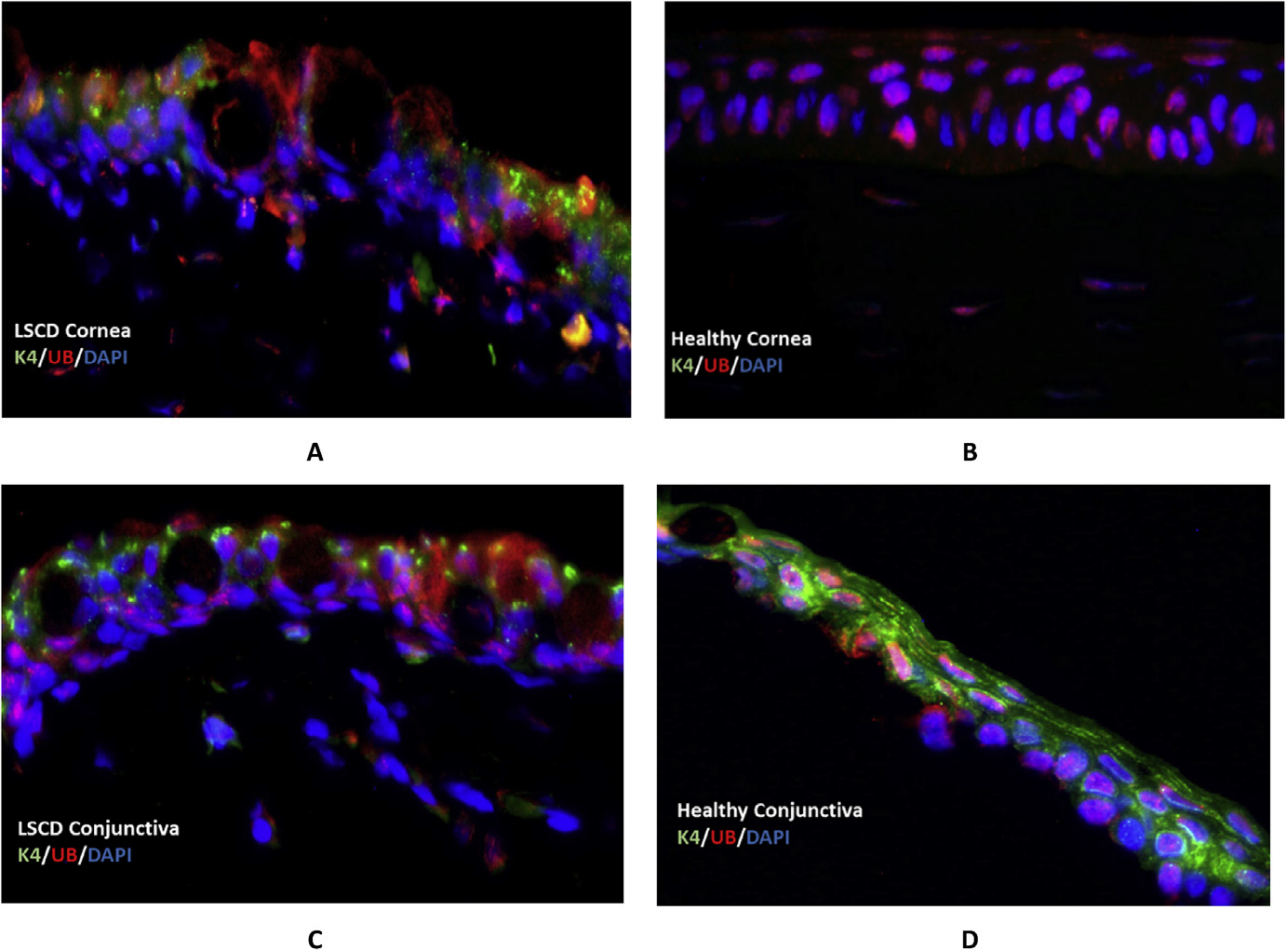
Double staining of LSCD-diseased and healthy rabbit corneal and conjunctival tissue sections with K4 (in green) and ubiquitin antibodies (in red). A: is an LSCD-diseased cornea and B: is a healthy cornea. C: is an LSCD-diseased conjunctiva and D: is a healthy conjunctiva. DAPI (blue) was used for nuclear stain, 100x).

We also conducted immunofluorescent double staining to specifically examine the expression of K4/K13 and ubiquitin in LSCD-diseased and healthy rabbit corneas. K4 expression was positive in LSCD corneas (Fig. 4A), while negative in healthy corneas (Fig. 4B). The expression of K4 showed an aggregate pattern as compared to healthy conjunctival K4 expression (Fig. 4C and D). The K4 positive cells also expressed ubiquitin in the cytoplasm while healthy corneas only expressed ubiquitin in the nucleus. Results also showed reproducible co-localization of K4 and ubiquitin in corneal epithelial cells of LSCD diseased ocular surface.

**Fig. 5:**
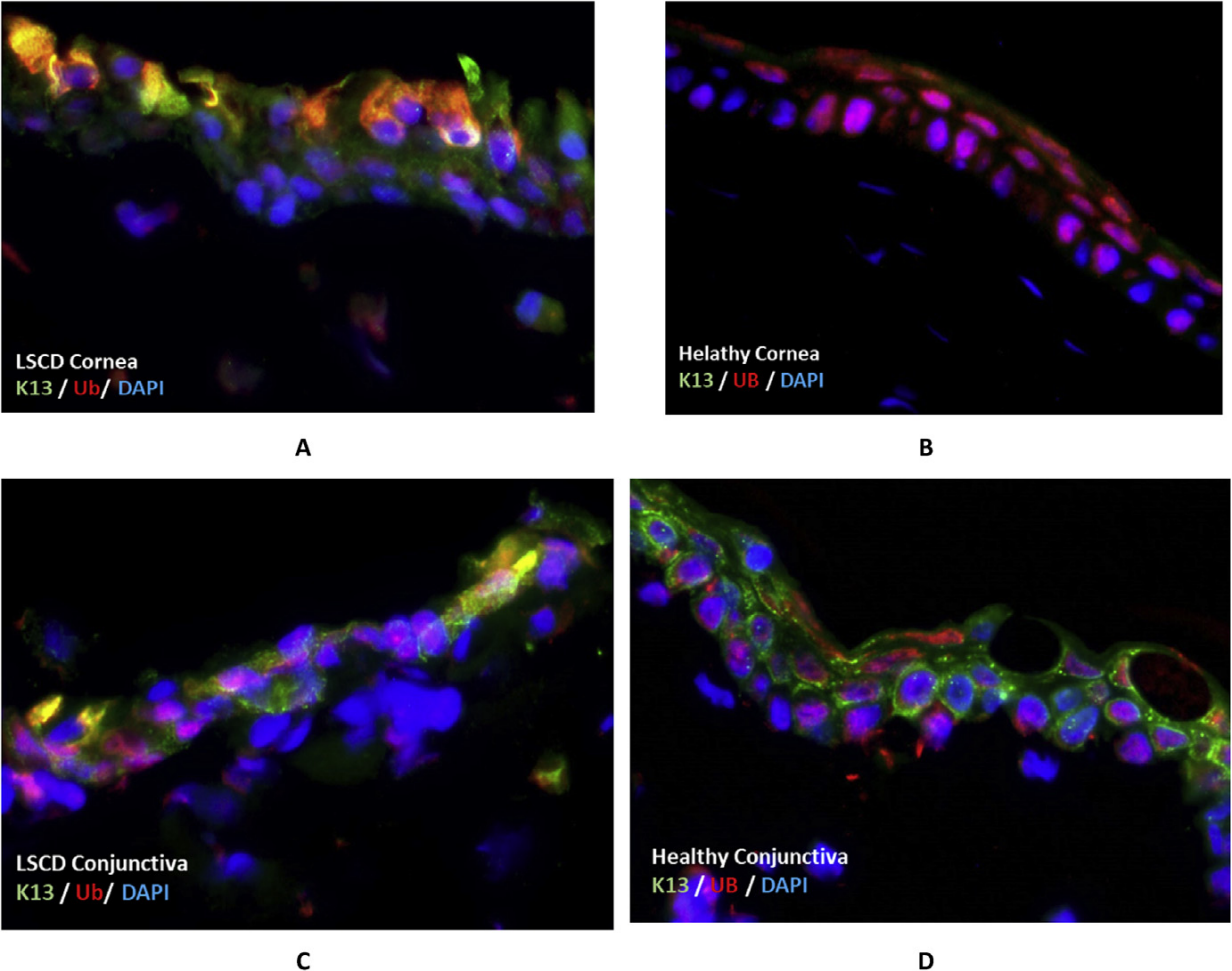
Double staining of LSCD-diseased and healthy rabbit corneal and conjunctival tissue sections with K13 (in green) and ubiquitin antibodies (in red). A: is an LSCD-diseased cornea and B: is a healthy cornea. C: is an LSCD-diseased conjunctiva and D: is a healthy conjunctiva. DAPI (blue) was used for nuclear stain. Magnification 100x.

Results also showed that K13 was markedly expressed in the cytoplasm of the outer corneal cells (Fig. 5). These cells also showed a marked expression of ubiquitin, indicating a co-localization of both proteins in the cytoplasm of these cells.

**Fig. 6:**
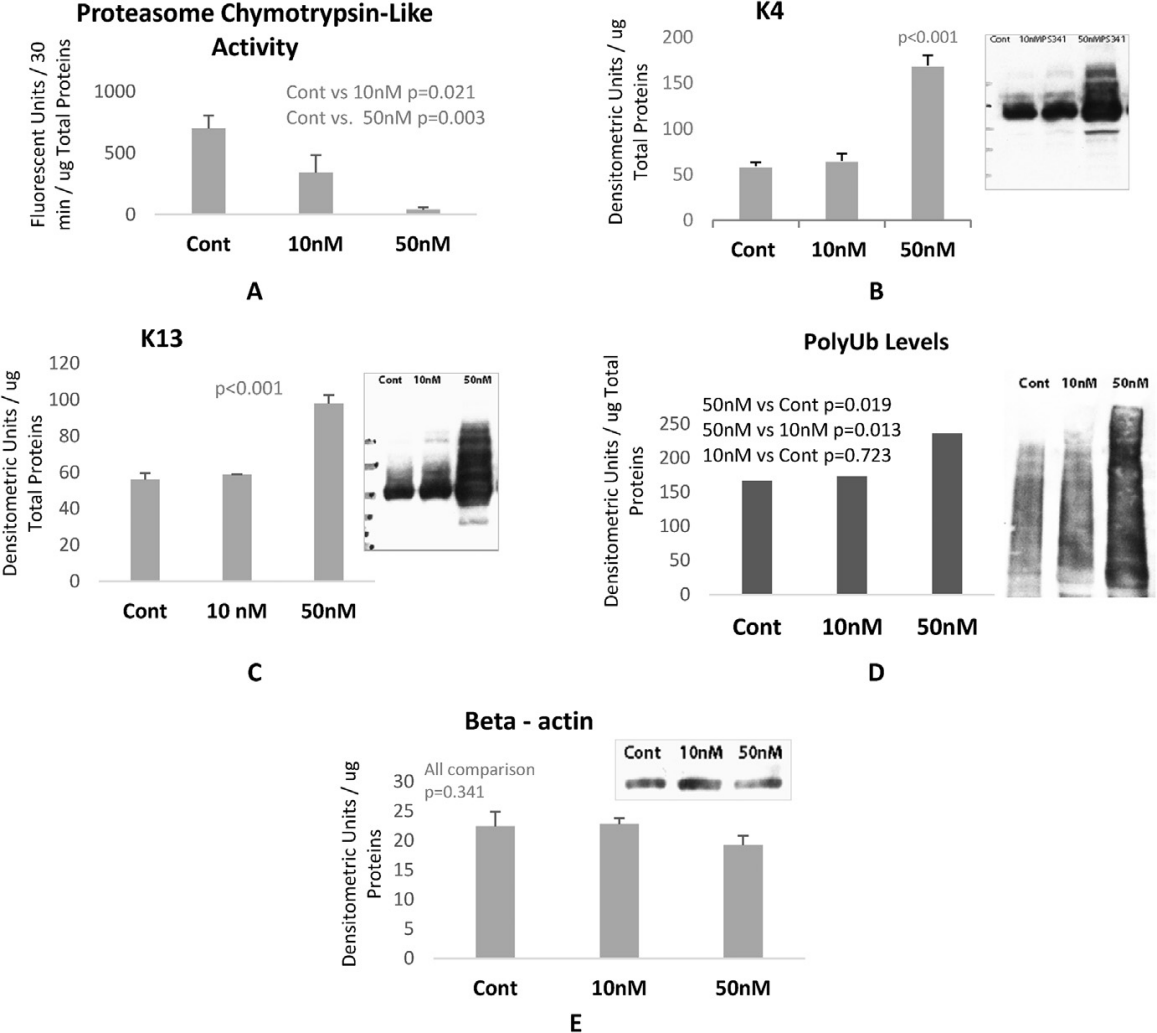
Oral mucosal epithelial cells (OMEC) were isolated, cultured and treated with high dose of proteasome inhibitor (PS341). A: Proteasome Chymotrypsine-like activity. B and C: are the measurement of K4 and K13 low molecular weight protein levels in OMEC treated with proteasome inhibitor. D: Poly ubiquitinated proteins levels were significantly high in cells treated with 50 nM of proteasome inhibitor. E: Beta-actin protein levels measurments used as a loading control.

To further substantiate the role of proteasome dysfunction in CKAGG formation, OMEC were cultured and treated with PS341 at high dose (10 and 50 nM) for 24 hours before cells harvest. K4 and K13 are a dominant and specific pair of keratins in OMEC [22, 23]. Fig. 6A shows that 10 nM and 50 nM doses of proteasome inhibitor caused 51% and 94% inhibition, respectively. Only the high dose of PS341 (50 nM) caused a significant accumulation of K4 and K13 (Fig. 6 B and C). Similar to LSCD corneal epithelial cells, OMEC treated with proteasome inhibitor showed an accumulation of keratins high molecular weight. This result indicates that K4 and K13 were modified and were not degraded because of proteasome failure. These results also support our hypothesis that there is a failure of modified keratin clearance when proteasome is inhibited.

**Fig. 7:**
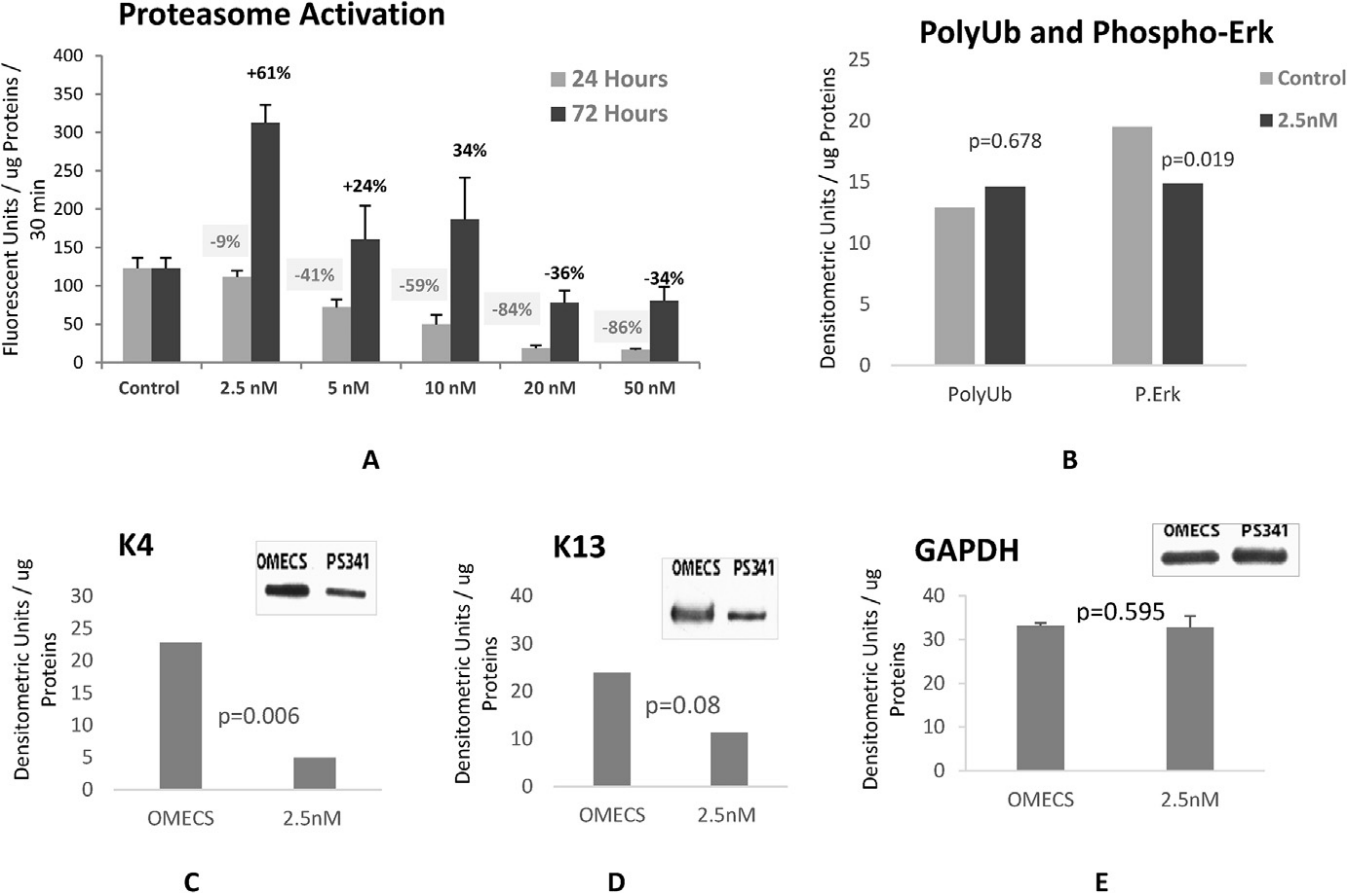
Proteasome activation lead to a significant decrease in K4 and K13 levels. A: Proteasome chymotrypsine-like activity. B: is the poly ubiquitinated proteins and phosporylated MAPKinase Erk protein levels examining the effects of proteasome inhibition. C and D: K4 and K13 levels in OMEC treated with proteasome inhibitor PS341 at 2.5 nM for 72 hours. E: Loading control examined by GAPDH levels analysis. Note that both K4 and K13 expression decreased in OMEC treated with proteasome activator.

Cells treated with 50 nM of proteasome inhibitor showed a marked accumulation of high molecular weight (HMW) of both keratins K4 and K13. These cells also showed a high level of polyubiquitinated proteins (Fig. 6 D). In order to increase K13 and K4 turn over, proteasome activation was investigated and accomplished by treating cultured cells with very low dose of proteasome inhibitor. OMEC were isolated and cultured for two weeks and treated with PS341 at 2.5, 5, 10, 20, and 50 nM for 24 h and 72 h (Fig. 6A). At 24 h, all PS341 doses lead to proteasome activity decrease (9%, -41%, -59%, -84% and -86%, respectively). However, 2.5 nM, 5 nM and 10 nM doses increased proteasome activities at 72 h. The highest increase in proteasome chymotrypsin-like activity was shown using 2.5 nM–61% higher than untreated cells (Fig. 7A).

As previously reported, the beneficial effects of proteasome inhibitor treatment at a very low dose of PS341 reside in the reversibility of the drug and in the rebound of proteasome activity 72 h after treatment [24]. Western blot analysis of cells sheets treated with 2.5 nM of PS341 for 72 h showed no significant polyubiquitinated protein accumulation and no apoptotic signal (Fig. 7B), indicating that this low dose treatment did not have any toxic effects. Results also showed that proteasome inhibition at this “homeopathic” dose did not cause accumulation of high molecular weight of modified K4 and K13 proteins (Fig. 7 C and D) and that there was a decrease in the levels of unmodified K4 and K13 proteins. The treatment with a low dose of proteasome inhibitor PS341 prevented the accumulation of modified K4 and K13. These results confirmed our hypothesis that proteasome activation may lead to a decrease of modified K4 and K13 levels in diseased epithelial cells.

## 4 Discussion

The goal of the present study was to investigate: 1) the accumulation of abnormal conjunctival keratins in central corneal epithelium after experimentally induced Limbal Stem Cell Deficiency (LSCD), and 2) the clearance of these accumulated keratins by proteasome activation. Both keratins K4 and K13 were found expressed in central cornea, even 3 months after limbectomy. LSCD corneal epithelial cells were analyzed and showed high molecular weight deposit of K4 and K13. In general, when LSCD is present, K4 and K13 expression is explained by the invasion of conjunctival cells toward central cornea. However, our results showed a different pattern in K4 and K13 expression in LSCD corneal epithelial cells when compared to healthy conjunctival cells (CjEC). We found a significantly higher amount of K4 and K13 and found extra bands with high molecular weight in LSCD corneal epithelial cells, as compared to healthy CjEC. The accumulation of these keratins, with high molecular weights, indicated a corneal keratin aggresomes formation (CKAGG). We believe that CKAGG is accumulated as the result of the LSCD-induced failure of the proteasome to clear damaged and deposited proteins. Constitutive proteasome activity was found significantly decreased in LSCD corneal epithelial cells [18], which was explained by the imbalance in proteasome population and the shift toward the formation of immunoproteasome to the detriment of constitutive proteasome responsible of damaged proteins clearance [18, 24]. It is therefore proposed that **both K4 and K13 are substrates for the ubiquitin proteasome pathway** (UPP) and that **this pathway regulates their levels**. There is no report in the literature documenting the clearance of damaged and otherwise altered K4 and K13 by the constitutive proteasome. The only keratins known to be degraded by the proteasome are K8 and K18 [25, 26, 27]. The latest two keratins were reported to be ubiquitinated and tagged to be digested by the proteasome [28, 29]. However, in case of pathological constitutive proteasome failure [30, 31, 32, 33], and severe proteasome inhibition [34], keratins K8 and K18 accumulated and aggregated to form, i.e., Mallory-Denk bodies in the liver. In corneal tissue sections, double staining of K4 or K13 with ubiquitin showed co-localization of K4 and K13 with ubiquitin. Because of LSCD-induced constitutive proteasome failure, keratins accumulated and deposited in insoluble aggregated (CKAGG) that might have trapped ubiquitin and other proteins involved in the process of aggregates clearance. Morphological analysis also showed less K13 aggregates as compared to K4 aggregates —with an impaired formation of keratin network that resulted in alteration in cell shape and attachment. K4 staining did not show a filamentous pattern as a normal corneal or conjunctival epithelial cells would have. Instead, K4 staining showed deposited aggregates, suggesting that abnormal expression of only one keratin subtype could cause an impaired cytoskeletal network. Based on the results of the present study, and the results of previous studies investigating K8/K18 [17, 35], we believe that the basic keratins (K4 and K8) are usually the proteins that undergo modification and cause aggregation to form intracellular bodies.

The popular view presents keratin aggresomes formations as harmless intracellular bodies deposited by the cell as temporary until the cell finds a way to clear this type of aggresomes. We believe that CKAGG formation might interfere with cornea transparency and contribute to vision loss. The disorganization of the corneal and stromal keratin filament network, and thus the cell structure, might compromise corneal transparency. The mechanism by which the ubiquitin proteasome pathway would digest K4 and K13 is not known yet. Understanding K4 and K13 clearance by proteasome is an important objective for corneal regeneration research and a significant advancement in ongoing research effort on cultured oral mucosal epithelial cell sheets for corneal epithelium reconstruction research because OMEC are also characterized by K4 and K13. OMEC were therefore used in our *in vitro* study to demonstrate the role of proteasome activity modulation in regulating the levels of K4 and K13. Previous findings [23, 36] have demonstrated the beneficial potential of a low dose of proteasome inhibitor, showing a rebound in proteasome chymotrypsin-like activity after 72 hours post treatment. Proteasome activation, applied for the first time to oral mucosal epithelial cells, showed a decrease in unmodified K4 and K13 expression and no deposition of keratin high molecular weight, indicating an active proteasome clearance of the unmodified forms. This concept could be applied to conjunctivalized ocular surface and possibly improve corneal transparency. Two experiments were conducted: **the first** experiment consisted of severely inhibiting OMEC proteasome activity with high doses of PS341. Results demonstrated that both modified and unmodified K4 and K13 deposits accumulated and that extra bands with high molecular weight appeared, as compared to untreated cells, which confirmed the results in LSCD-diseased CEC as that CKAGG formation occurred when there is UPP failure. **The second** experiment consisted of activating OMEC proteasome with very low doses of PS341. Results demonstrated that both unmodified K4 and K13 expressions were decreased and there was no keratin high molecular weight deposition when proteasome activation was achieved.

Several cellular disorders are related to proteasome inhibition or proteasome failure. To date, proteasome activation has not been associated with any detrimental cellular function. We believe that proteasome activation in the ocular surface would be beneficial to alleviate LSCD symptoms, possibly improving corneal transparency after injury. Proteasome activation was previously achieved in rat liver [23, 36]. The beneficial effects of proteasome activation translated into an up-regulation of heat shock proteins that prevent protein aggregates formation [26]. It also translated into an up-regulation of anti-oxidative elements, thus blocking the insults-induced oxidative stress in liver cells [37], and into a decrease in key enzymes involved in cholesterol syntheses and intestinal fat adsorption [37]. The beneficial effects of proteasome activation were also demonstrated when PS341 was supplemented in the solution of preservation prior to organ transplantation [38, 39, 40, 41].

The mechanism by which a low dose of PS341 induces proteasome activation is interesting because it mildly inhibits proteasome activity during the first 24 h and subsequently the cells *sense* it and possibly react by activating the assembly of already synthesized proteasome subunits to make more proteasome 72 h after mild inhibition. We did not find an increase in proteasome alpha and beta subunits 72 hours after mild inhibition, which lead us to think that there is possibly an assembly process activation [42].

Despite current treatments involving cell-based therapy or VEGF pharmacological approaches, cornea conjunctivalization is still not well managed. Almost all subsequent research and clinical advances have been made with proteasome inhibitors. This, in turn, put a shadow on the beneficial aspect of proteasome activation. Currently, there are no commercially available proteasome activators.

In conclusion, the present study demonstrates the existence of keratin aggregation in LSCD corneal epithelial cells and points out the role of proteasome failure in the formation of CKAGG. CKAGG were mainly found in the epithelium of LSCD-diseased cornea, suggesting that CKAGG formation may play a contributing role in LSCD -induced corneal epithelial cells cloudiness. The long-term goal of this research is to investigate whether abnormal expression of keratin contributes to corneal epithelial cells cloudiness, and how proteasome activation could alleviate CKAGG formation.

## Declarations

### Author contribution statement

Fawzia Bardag-Gorce: Conceived and designed the experiments; Performed the experiments; Analyzed and interpreted the data; Contributed reagents, materials, analysis tools or data; Wrote the paper.

Andrew Makalinao, Imara Meepe, Daileen Cortez, Amanda Laporte, Jeremy Stark, Amber Gorce: Performed the experiments.

Richard H. Hoft, Joan Oliva, Michael Di Lorenzo: Performed the experiments; Analyzed and interpreted the data.

Samuel W. French: Analyzed and interpreted the data; Contributed reagents, materials, analysis tools or data.

William Lungo: Performed the experiments; Contributed reagents, materials, analysis tools or data.

Yutaka Niihara: Analyzed and interpreted the data.

### Funding statement

This work (salaries and research) was supported by Emmaus Medical, Inc.

### Competing interest statement

The authors declare the following conflict of interest: salaries and research were supported by Emmaus Life Sciences Inc.

### Additional information

No additional information is available for this paper.

## Figures and Tables

**Supplementary figure to figure 2D:**
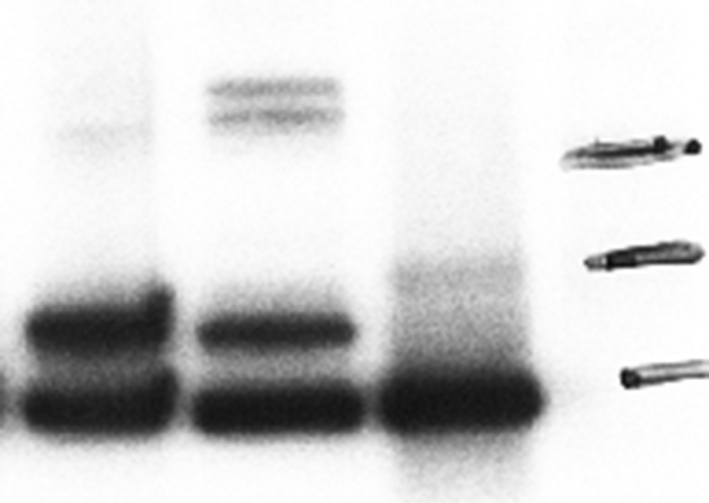


**Supplementary figure to figure 6E:**
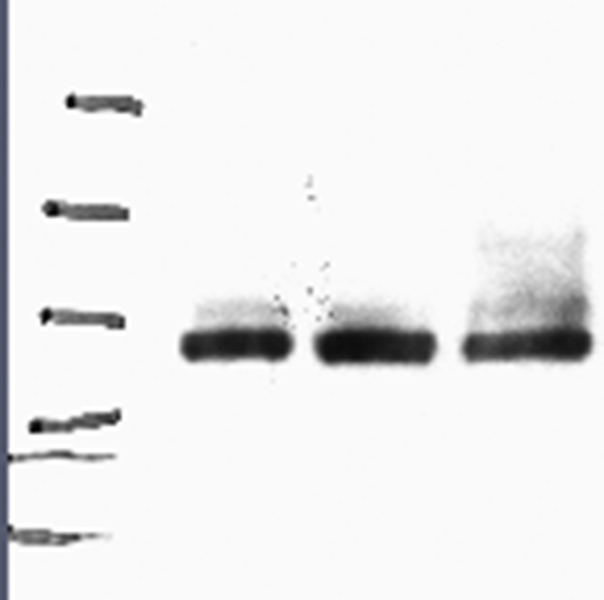


**Supplementary figure to figure 7C:**
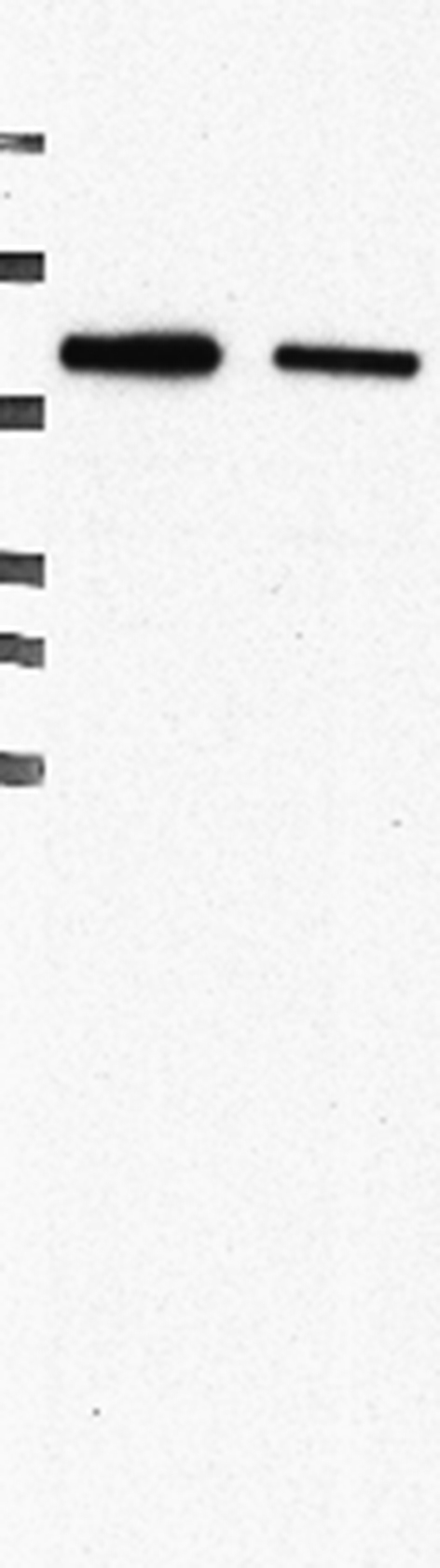


**Supplementary figure to figure 7D:**
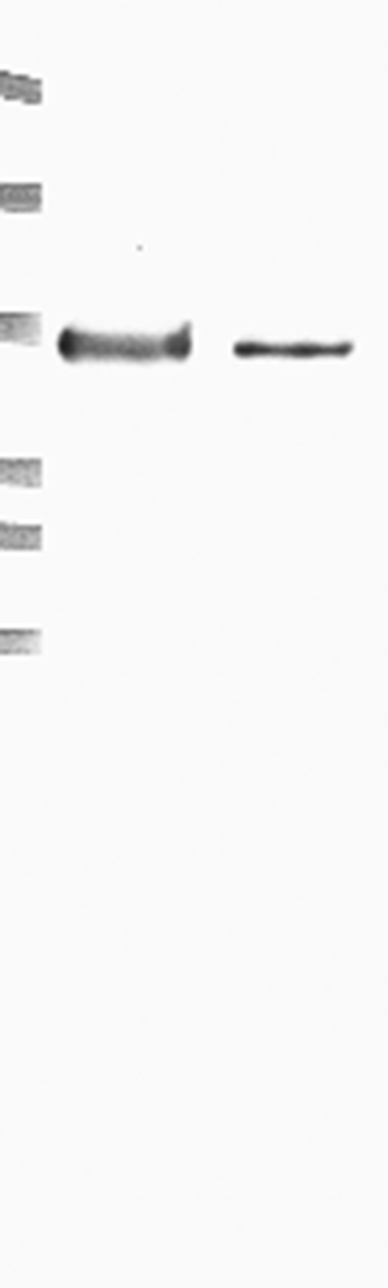


**Supplementary figure to figure 7E:**